# Bibliography

**Published:** 1889-11

**Authors:** 


### BIBLIOGRAPHY.

Annual of the Universal Medical Sciences: A Yearly Report of the Progress of the General Sanitary Sciences throughout the World. Edited by Charles E. Sajous, M.D., and Seventy Associate Editors, assisted by over two hundred Correspondents and Collaborators. Five vols., 8vo. Numerous Chromo-Lithographic Illustrations, Engravings, and Maps. 1889. $15 per annum. F. A. Davis, Publisher, Philadelphia, New York, and London.

The second edition of this valuable work has been delivered, and met with universal favor at the hands of the profession. Many improvements were made in the arrangement of the text and the mechanical execution of the work. To each reference has been added the date, number, or volume of the journal quoted. Foreign weights and measures have been reduced for the benefit of those who do not understand them; a complete and compact index has been added to each volume. The annual stands to-day without a peer as a reference hand-book of the latest advances made in every department of scientific research, and should be in the library of every dentist who has any aspirations to possess a library and do any reading outside of his own specialty. Short, comprehensive reviews of the best articles printed in all languages are therein found. We are sorry to say that the section on dentistry has been discontinued, but it was found impracticable to carry it on, as not enough dentists showed an inclination to encourage its continuation.

Human Physiology. By Landois and Stirling. With 692 Illustrations. Third American, from the Sixth German Edition. A Text-Book of Human Physiology, including Histology and Microscopical Anatomy, with special reference to the requirements of Practical Medicine. By Dr. L. Landois, Professor of Physiology and Director of the Physiological Institute, University of Greifswald. Translated from the Fifth German Edition, with additions by Wm. Stirling, M.D., Sc.D., Brackenburg, Professor of Physiology and Histology in Owen's College and Victoria University, Manchester; Examiner in the Honors' School of Science, University of Oxford, England. Second Edition, revised and enlarged. 692 Illustrations. "A bridge between Physiology and Practical Medicine." One Volume. Royal Octavo. Cloth, $6.50; leather, $7.50.

"*From the Prefaces to the English Edition.*—'The fact that Professor Landois's book has passed through four large editions in the original since 1880, and that in barely six months' time a second edition of the English has been called for, shows that in some special way it has met a want. The characteristic which has thus commended the work will be found mainly to lie in its eminent practicability; and it is this consideration which has induced me to undertake the task of putting it into English. Landois's work, in fact, forms a *Bridge* between Physiology and the Practice of Medicine. It never loses sight of the fact that the student of to-day is the practising physician of to-morrow. In the same way, the work offers to the busy physician in practice a ready means of refreshing his memory on the theoretical aspects of medicine. He can pass backward from the examination of pathological phenomena to the normal processes, and, in the study of these, find new indications and new lights for the appreciation and treatment of the cases under consideration. With this object in view, all the methods of investigation which may, to advantage, be used by the practitioner are carefully and fully described. Many additions, and about one hundred illustrations, have been introduced into this second English edition, and the whole work carefully revised.'"

After a careful perusal, we can fully recommend Professor Landois's physiology to students as a standard work on the subject. It is especially noticeable for the practical every-day manner in which all subjects are treated. Its conciseness is a marvel when its completeness is taken into consideration.

We are in receipt of two more copies of the popular "Physicians' Leisure Library" series. Published by Geo. S. Davis, Detroit, Michigan. Single copies, 25 cts. each.

The first relates to the treatment of the morphia habit, by Albrecht Erlenmeyer, and translated by E. P. Hurd, M.D. It is a practical treatise, giving methods of treatment, citing cases, and is of particular interest to physicians only; but the other volume, on *Dyspepsia*, by Frank Woodbury, M.D., is of special interest to dentists; for who in our profession are not more or less subject to this malady. Dr. Woodbury handles the subject in a clear and concise manner, taking up the cause, symptoms, and treatment.

Orthodontia; or, The Malposition of the Human Teeth: Its Prevention and Remedy. By S. H. Guilford, A.M., D.D.S., Ph.D., Professor of Operative and Prosthetic Dentistry in the Philadelphia Dental College; Author of "Nitrous Oxide," etc. Press of Spangler & Davis, Philadelphia; and for sale by the S. S. White Dental Manufacturing Co.

The finished work was submitted to the National Association of Dental Faculties at its last meeting.

The first fifty-five pages are devoted to the principles involved in the art. Part II., fifty-one pages, considers materials and methods; and Part III. takes up specific forms of irregularities and their treatment. The book is well gotten up, is amply illustrated, and presents a very creditable appearance.

It comes nearer being the ideal text-book than any that have yet been presented to the association for their approval. In it a student may find principles discussed to a sufficient extent to enable him to understand the methods employed, and the ample illustrations used make their application easy to understand. It should be in the hands of every student.

AIDE-MÉMOIRE DU CHIRURGIEN-DENTISTE. Par M. PAUL DUBOIS. Directeur de l'Odontologie; Président de la Société d'Odontologie de Paris; Professeur Suppléant de Thérapeutique spéciale à l'École Dentaire de Paris. Deuxième Édition. Première Partie, Thérapeutique de la Carie Dentaire. Livre deuxième. Dentisterie Opératoire, Liée au Traitement de la Carie Dentaire.

This is a neat-appearing treatise on Operative Dentistry by the editor of "L'Odontologie." The first two hundred pages are devoted to the pathology and therapeutics of dental caries. The author divides decay into four classes or degrees, as he styles them, and considers each separately, a very admirable method indeed. The remainder of the book is given to Operative Dentistry, including filling, pivoting, bridge-work, implantation, extraction, and anæsthesia. We may congratulate Prof. DuBois on the mechanical execution of his book as well as the context, which is exceedingly well systematized, and shows the author to be well posted upon the subjects.

DIE MICROORGANISMEN DER MUNDHÖHLE is the title of Dr. W. D. MILLER'S book, recently issued in German. It is gotten up in the style so common in Germany, being unbound and uncut. It contains three hundred pages, and is replete with information regarding the micro-organisms found in the mouth. Much of the text has already appeared in the INDEPENDENT PRACTITIONER, and is familiar to its readers, especially the portions directly relating to decay of the teeth. Several chapters have, however, been added on the general subject of micro-organisms and the whole thoroughly rewritten. We understand that the S. S. White Dental Manufacturing Company have assumed the responsibility of its translation and issue in English. We would advise our readers to purchase it, as it will form a complete volume of the researches of Dr. Miller to date.

It is not our custom to notice catalogues in our reading columns, but the one received from the S. S. White Dental Manufacturing Company on porcelain teeth is such an exception to the ordinary catalogue that we forbear the rule. It contains one hundred and fifty pages, the first fifty of which are devoted to a scientific presentation of the law of correspondence and temperament in relation to the selection of artificial teeth. The whole forms a very instructive treatise on the subject of prosthetic dentistry, as far as it relates to the selection of artificial teeth, which is the most important part of the art, for without a complete knowledge of the temperamental aspect of the question no prosthetic dentist can consider his education complete. There are but few men in the profession who would not be benefited by a careful study of the catalogue, for it is replete with information from cover to cover.

We are also in receipt of a number of reports of society proceedings and pamphlets,—to wit:

Proceedings of the Chicago Dental Society, 1889; Transactions of the Odontological Society of Pennsylvania, 1886, 1887, 1888; Proceedings of the New Jersey State Dental Society, 1888; Transactions of the Pennsylvania State Dental Society, 1888. The Constitutional Treatment of Caries and Necrosis, and Diastatic Food in the Treatment of Chronic Diseases and Deformities of the Bones of Children, by Hal C. Wyman, M.S., M.D., Detroit; Cysts of the Jaw, with the Report of a Case, by J. L. Gist, M.D., Jackson, Michigan.

